# Paediatric/young versus adult patients with long QT syndrome

**DOI:** 10.1136/openhrt-2021-001671

**Published:** 2021-09-12

**Authors:** Sharen Lee, Jiandong Zhou, Kamalan Jeevaratnam, Wing Tak Wong, Ian Chi Kei Wong, Chloe Mak, Ngai Shing Mok, Tong Liu, Qingpeng Zhang, Gary Tse

**Affiliations:** 1 Cardiovascular Analytics Group, Hong Kong, China-UK Collaboration; 2 School of Data Science, City University of Hong Kong, Hong Kong, People's Republic of China; 3 Faculty of Health and Medical Sciences, University of Surrey, Guildford, Surrey, UK; 4 School of Life Sciences, Chinese University of Hong Kong, Hong Kong, People's Republic of China; 5 Research Department of Practice and Policy, University College London School of Pharmacy, London, UK; 6 Department of Pathology, Hong Kong Children's Hospital, Hong Kong, People's Republic of China; 7 Department of Medicine and Geriatrics, Princess Margaret Hospital, Hong Kong, People's Republic of China; 8 Tianjin Key Laboratory of Ionic-Molecular Function of Cardiovascular Disease, Department of Cardiology, Tianjin Institute of Cardiology, Second Hospital of Tianjin Medical University, Tianjin, People's Republic of China

**Keywords:** arrhythmias, cardiac, tachycardia, ventricular, ventricular fibrillation

## Abstract

**Introduction:**

Long QT syndrome (LQTS) is a less prevalent cardiac ion channelopathy than Brugada syndrome in Asia. The present study compared the outcomes between paediatric/young and adult LQTS patients.

**Methods:**

This was a population-based retrospective cohort study of consecutive patients diagnosed with LQTS attending public hospitals in Hong Kong. The primary outcome was spontaneous ventricular tachycardia/ventricular fibrillation (VT/VF).

**Results:**

A total of 142 LQTS (mean onset age=27±23 years old) were included. Arrhythmias other than VT/VF (HR 4.67, 95% CI (1.53 to 14.3), p=0.007), initial VT/VF (HR=3.25 (95% CI 1.29 to 8.16), p=0.012) and Schwartz score (HR=1.90 (95% CI 1.11 to 3.26), p=0.020) were predictive of the primary outcome for the overall cohort, while arrhythmias other than VT/VF (HR=5.41 (95% CI 1.36 to 21.4), p=0.016) and Schwartz score (HR=4.67 (95% CI 1.48 to 14.7), p=0.009) were predictive for the adult subgroup (>25 years old; n=58). A random survival forest model identified initial VT/VF, Schwartz score, initial QTc interval, family history of LQTS, initially asymptomatic and arrhythmias other than VT/VF as the most important variables for risk prediction.

**Conclusion:**

Clinical and ECG presentation varies between the paediatric/young and adult LQTS population. Machine learning models achieved more accurate VT/VF prediction.

**Key questions**

**What is already known about this subject?**

- Long QT Syndrome (LQTS) is an ion channelopathy that predisposes affected patients to spontaneous ventricular tachycardia/fibrillation (VT/VF) and sudden cardiac death. However, differences in epidemiology and risk factors between the paediatric/young and adult subgroups within the Chinese patient population are not well-defined.

**What does this study add?**

- There are significant differences in clinical and electrocardiographic presentation amongst adult and paediatric/young LQTS patients.
- Adult LQTS patients have a higher risk for spontaneous VT/VF.
- Similar predictors were found in the overall LQTS cohort and adult subgroup.
- A nonparametric machine learning survival analysis can achieve much higher accuracy to predict the incident VT/VF probabilities of LQTS patients through accounting for the interactions between predictors.

**How might this impact on clinical practice?**

- Clinical and ECG presentation of LQTS vary between the paediatric/young and adult LQTS population. Risk stratification and management strategies against young patients should take into consideration the difference between paediatric and adult patients and adopt an individualised approach. Machine learning models achieved more accurate VT/VF prediction.

## Introduction

Cardiac ion channelopathies predispose to the development of spontaneous ventricular tachycardia/fibrillation (VT/VF) potentially leading to sudden cardiac death (SCD),^1 2^ and other cardiac rhythm disturbances.^3–5^ Of these, long QT syndrome (LQTS) is a less prevalent condition compared with Brugada syndrome (BrS) in Asia.^6 7^ In LQTS, the characteristic feature is ECG QT prolongation,^8–11^ reflecting delayed repolarisation at the cellular level.^12 13^

The age of presentation differs between the different cardiac ion channelopathies. Among the current 17 LQTS subtypes,^14^ the most prevalent type 1 LQTS usually presents in late childhood, while type 2 patients have the highest risk of VT/VF during the 9 months post partum.^6^ It should also be noted that recent works have suggested that the majority of rare genetic causes have, at best, a weak genotype–phenotype association.^15^ Due to the fewer number of paediatric LQTS patients, it can be challenging to identify the specific differences between the paediatric and adult subgroups. As a result, the application of adult-based risk stratification criteria on the paediatric population may result in misinterpretation of SCD risk. This is an important clinical issue because invasive treatment such as the insertion of implantable cardioverter-defibrillators, despite effectiveness in preventing arrhythmic events, is associated with complications and problems such as inappropriate shocks.^16^ Therefore, the present study aims to demonstrate the difference in clinical and ECG presentation between paediatric/young and adult LQTS patients.

## Methods

### Study population

The cohort included consecutive patients diagnosed with LQTS between 1 January 1997 and 20 June 2020, in public hospitals of Hong Kong. Centralised electronic health records from the Hospital Authority were reviewed for patient identification and data extraction. This system has previously been used by our team to study the epidemiology and outcomes of ion channelopathies such as BrS.^17–20^ The diagnoses of the respective ion channelopathies were made initially by the case physicians. They were confirmed by GT and NSM through the review of case notes, documented ECGs, diagnostic test results, and genetic reports. Diagnosis of LQTS was made if the disease was not induced by drugs, hypokalaemia or hypomagnesaemia and fulfil one of the following: (1) Schwartz score greater or equal to 3.5; (2) positive for LQTS-related mutations on genetic testing and (3) initial QTc interval greater than 500 ms. All LQTS patients, except for one Japanese immigrant, were Han Chinese.

### Clinical and electrocardiographic data collection

The baseline clinical data extracted from the electronic health records include: (1) sex; (2) age of first characteristic ECG presentation and last follow-up; (3) follow-up duration; (4) family history of SCD and the specific ion channelopathy; (5) syncope manifestation and its frequency; (6) presentation of sustained VT/VF and its frequency; (7) performance of electrophysiological study (EPS), 24-hours Holter study, ion channelopathy-specific genetic testing, and the respective results; (8) performance of echocardiogram; (9) presence of other arrhythmias; (10) implantation of implantable cardioverter-defibrillator (ICD); (11) occurrence, cause and age of death; (12) period between the initial presentation of characteristic ECG and the first postdiagnosis VT/VF episode and (13) initial disease manifestation (asymptomatic, syncope, VT/VF). In the present study, symptoms refer to syncope or VT/VF, thus asymptomatic indicates freedom from either presentation. Other arrhythmias include sick sinus syndrome, bradycardia, atrioventricular block, atrial tachyarrhythmias and supraventricular tachyarrhythmias. Positive EPS is defined as the induction of spontaneous VT/VF that either sustained a minimum of 30 s or produced haemodynamic collapse.

The following additional information was extracted: (1) type of LQTS; (2) QTc interval at the initial presentation of QTc prolongation; (3) performance and the result of the treadmill test, with a positive test defined as the presence of exercise-recovery-induced QT prolongation and (4) Schwartz score. It should be noted that the genotype–phenotype association in rare genetic causes can be weak, thus, the type of LQTS is noted for documentation purposes. A positive result in the treadmill test is defined as the presence of exercise-recovery-induced QT prolongation, ST depression, syncope or VT/VF during the test. Schwartz’s score was calculated based on the clinical and ECG data documented in case records using the original definitions^21^:

1. ECG criteria: QTc by Bazett’s formula ≥480 ms (three points), =460–479 ms (two points), =450–459 ms (males) (one point) and ≥480 ms during fourth minute of recovery from exercise stress test (one point), torsades de pointes (two points), T-wave alternans (one point), notched T-wave in three leads (one point), low heart rate for age (0.5 points).
2. Clinical criteria: syncope with stress (two points), syncope without stress (one point).
3. Family history: family history with definite LQTS (one point), unexplained SCD at age <30 years in the immediate family (0.5 points).

The following automated measurements were extracted from baseline ECGs: (1) heart rate; (2) P wave duration (PWD) and PR interval (duration between onset of P-wave and onset of R-wave); (3) QRS duration (duration between onset of Q-wave and end of S-wave); (4) QT and QTc interval; (5) P, QRS and T wave axis; (6) amplitude of R and S wave from leads V5 and V1 respectively; (7) presence of first degree atrioventricular block, defined as PR interval greater than 200 ms and (8) presence of interventricular delay, defined as QRS-interval greater or equal to 110 ms. Baseline ECG is the documented ECG taken at or the earliest after the initial characteristic ECG presentation, hence, the patient was not given any therapy at the time. All ECG parameters, except for the amplitude of R-wave and S-wave from leads V5 and V1, respectively, were averaged across the 12 leads.

### Classification and statistical analysis

The study population is classified into paediatrics and adult based on the age of onset for the ion channelopathy-characteristic ECG. Patients were divided into paediatric/young (less than or equal to 25 years old) and adult (more than 25 years old) subgroups.^22^ Subgroup differences of categorical variables were compared through Fisher’s exact test and reported as total number (percentage), while discrete and continuous variables were compared by Kruskal-Wallis one-way analysis of variance and expressed as mean±SD. The mean annual VT/VF incidence rate of each subgroup is calculated by first obtaining the patient-specific rate through dividing the total number of sustained VT/VF events by the follow-up period, then average the rates within the subgroup. Statistical significance is defined as p<0.05. The difference in the duration of postdiagnosis VT/VF-free survival between the paediatric and adult subgroup is compared quantitatively by both the log-rank test and Cox proportional HR regression, and qualitatively by Kaplan-Meier survival curve.

Both univariate and multivariate Cox regression was used to identify independent predictors for a shorter time to first postdiagnosis sustained VT/VF. The HR and 95% CI are reported for Cox regression. Due to the limited VT/VF event within the paediatric subgroup, Cox regression was performed for the overall cohort and the adult subgroup. A maximum of five univariate predictors with the greatest statistical significance, at least p<0.10, were selected for the multivariate analysis to avoid overfitting. The included univariate predictors must be unrelated and independent to each other. Separate models with and without the inclusion of predictors from the baseline ECG were established. All statistical analysis was performed using R Studio (V.1.3.1073).

### Development of a machine learning survival analysis model

Survival analysis models are used to predict the risk of future time-to-events. Commonly, the default choice is Cox regression because of its convenience. Random survival forest (RSF) is a class of survival analysis models that use data on the life history of patients (the outcome or response) and their meaningful characteristics (the predictors or variables).^23^ RSF extends the traditional random forests algorithm for a target which is not a class, or a number, but a survival curve. RSF is non-parametric and does not assume proportional risks as in the Cox model. This allows direct learning of the survival patterns between predictors and outcome. RSF bypasses the traditional necessity to impose parametric or semiparametric assumptions on the underlying distributions of censored data, and therefore, provides an alternative approach to automatically deal with high-level interactions and higher-order non-linear terms in variables and achieve much higher accurate survival predictions. The time-to-event prediction task through the RSF model aims to characterise the covariate effects on the time of a future VT/VF event, while capitalising on meaningful information from censored data when performing learning.

Significant univariate predictors identified by the Cox regression model are used as candidate inputs of the RSF model to predict primary VT/VF outcome. This avoided possible collinearity and overfitting that may occur if all predictors were included. A variable importance ranking approach was adopted based on standard bootstrap theory to investigate the strength of the associated significant univariate variables to predict VT/VF. Out-of-bag (OOB) method was adopted whenever a bootstrap sample is down with replacement from the training dataset. The importance value for the variable of interest was calculated as the prediction error (squared loss) for the original ensemble event-specific cumulative probability function (obtained when each OOB instance is just dropped down its in-bag competing risks tree) subtracted from the prediction error for the new ensemble obtained using randomising assignments of the variable.^24^ Variables that were important predictors of VT/VF has a larger importance value, indicating higher predictive strength, whereas non-predictive variables have zero or negative values.

A fivefold cross-validation approach was used to compare the survival prediction performance of the RSF model with the multivariate Cox regression model (both with significant univariate predictors as input). Evaluation measures of precision, recall, Brier score and rank statistics of Harrell’s C-index are used to assess the resulting probabilistic risk prediction comparisons. Training and testing of the RSF model for predicting VT/VF were conducted using the ggRandomForests R package. Survival estimates were calculated using the Brier score (0=perfect, 1=poor and 0.25=guessing) based on the inverse probability of censoring weight (IPCW) method.^25^ The cohort was stratified into four groups of 0–25, 25–50, 50–75 and 75–100 percentile values of VT/VF.

## Results

### Baseline characteristics

The cohort consisted of 142 consecutive patients (mean onset age=27±23 years old; female=60%; mean follow-up period=98±65 months; initial QTc interval=504±44 ms), and divided into adult (n=58; mean onset age=50±16; female=66%; mean follow-up period=95±67 months; initial QTc interval=507±51 ms) and paediatric/young subgroup (n=84, mean onset age=11±7 years; female=56.0%; mean follow-up period=91±64 months; initial QTc interval=501±38 ms) (table 1). A total of 100 patients were unrelated probands. The remaining 42 patients were from 16 families. There were 15 patients that were included based on LQTS. There is no significant intergroup difference in patients’ sex (p=0.298), follow-up duration (p=0.743) and initial QTc interval (p=0.510). Among patients with identifiable subtypes of LQTS, the present cohort consists of: type 1 (n=32), type 2 (n=29), type 3 (n=7), type 5 (n=1), type 8 (n=2), type 9 (n=1), type 16 (n=1). The subgroup differences for family history of LQTS (p=0.601) and SCD (p=0.166) were insignificant.

**Table 1:** Baseline characteristics of the cohort

Characteristic	Overall (n=142)	Adult (n=58)	Paediatric/young (n=84)	P value
Clinical characteristics				
Female	85 (59.9)	38 (65.5)	47 (56.0)	0.298
Onset age	27±23	50±16	11±7	**<0.0001**
Current age	34±23	58±16	18±10	**<0.0001**
Initial QTc interval	504±44	507±51.2	501±38	0.510
Family history of LQTS	56 (39.4)	21 (36.2)	35 (41.7)	0.601
Family history of SCD	22 (15.5)	12 (20.7)	10 (11.9)	0.166
Initial asymptomatic	63 (44.4)	24 (41.4)	39 (46.4)	0.608
Initial syncope	46 (32.4)	13 (22.4)	33 (39.3)	**0.045**
Initial VT/VF	33 (23.2)	21 (36.2)	12 (14.3)	**0.004**
Syncope	61 (43.0)	24 (41.4)	37 (44.0)	0.863
Syncope frequency	0.96±1.44	0.81±1.25	1.06±1.56	0.519
VT/VF	52 (36.6)	32 (41.4)	20 (23.8)	**<0.001**
Sustained VT/VF frequency	2.3±13.0	2.6±9.7	2.1±14.9	**<0.001**
Annual VT/VF incidence rate	0.6±2.6	0.6±1.6	0.5±3.1	**<0.001**
EPS	6 (4.2)	3 (5.2)	3 (3.6)	0.688
Positive EPS	4 (66.7)	2 (66.7)	2 (66.7)	1.00
ICD	51 (35.9)	33 (56.9)	18 (21.4)	**<0.0001**
Holter	53 (35.9)	14 (24.1)	39 (46.4)	**0.008**
Arrhythmia in holter study	33 (62.3)	11 (78.6)	22 (56.4)	0.336
Other arrhythmias	29 (20.4)	13 (22.4)	16 (19.0)	0.675
Genetic test	92 (64.8)	23 (39.7)	69 (82.1)	0.069
Positive genetic test	77 (83.7)	19 (82.6)	58 (84.1)	1.00
Treadmill test	48 (33.8)	6 (10.3)	42 (50.0)	**<0.0001**
Positive treadmill test	33 (68.8)	3 (50.0)	30 (71.4)	0.112
Schwartz score	4.24±1.13	4.20±1.11	4.27±1.15	0.723
Death	9 (6.34)	7 (12.1)	2 (2.38)	**0.032**
Follow-up duration	97.7±65.0	95.2±67.3	91.1±63.7	0.743
Baseline ECG characteristics				
Heart rate	76±24	70±19	82±27	**0.041**
P-wave duration	104±16	110±20	99±9	**0.019**
PR interval	161±30	169±28	155±30	**0.005**
QRS interval	97±22	104±26	90±15	**0.004**
QT interval	445±70	462±57	432±79	**0.024**
QTc interval	489±44	492±45	487±44	0.438
P Axis	55±41	66.7±49	44±30	**0.005**
QRS axis	56±59	48±73	65±39	**0.012**
T axis	53±55	68.1±69	39±32	0.090
Lead V5 R wave amplitude	1.17±0.65	1.15±0.82	1.18±0.42	0.361
Lead V1 S wave amplitude	0.70±0.42	0.65±0.42	0.74±0.42	0.298
First degree AV block	7 (4.9)	5 (8.6)	2 (2.4)	0.236
Interventricular delay	17 (12.0)	13 (22.4)	4 (4.8)	**0.015**

Bold values indicate P<0.05.

AV, atrioventricular; EPS, electrophysiological study; ICD, implantable cardioverter-defibrillator; LQTS, long QT syndrome; VT/VF, ventricular tachycardia/ventricular fibrillation.

In terms of disease manifestation, an initial presentation with syncope was more common in the paediatric/young compared with the adult patients (39% vs 22%), whereas initial presentation of VT/VF was more common in adults than paediatric/young patients (36% vs 14%). The adult subgroup was significantly more likely to develop VT/VF (p<0.001), with a greater frequency of sustained VT/VF (p<0.001) and mean annual VT/VF incidence rate (adult=0.632±1.61 VT/VF per year, paediatric/young=0.502±3.13 VT/VF per year, p<0.001), which contributed to a greater proportion of adult patients with ICD implanted (p<0.0001) and all-cause mortality (p=0.032). Among the paediatric population, arrhythmic events occurred either during the period of antiarrhythmic dosage titration, under a state of hypokalaemia or postoperatively. Throughout the follow-up period, there is only one case of SCD in the present cohort. The remaining eight cases of death are due to non-cardiovascular causes. Twenty-four hours Holter study (p=0.008) and treadmill test (p<0.0001) were more commonly performed among the paediatric/young subgroup, but the proportion of patients with positive findings did not differ significantly (arrhythmia in Holter study: p=0.336; positive treadmill test: p=0.112). Arrhythmia in Holter study includes VT (n=7), ventricular ectopics/premature ventricular complex (n=21), supraventricular tachycardia/ectopics (n=17) and conduction defects (n=4). Different types of arrhythmia can appear in the Holter study of the same patient. Out of the 48 LQTS patients that have undergone exercise stress tests, 33 had positive tests.

In terms of baseline ECG indices, the adult subgroup had significantly higher value in the following parameters: (1) PWD (p=0.019); (2) PR interval (p=0.005); (3) QRS interval (p=0.004); (4) QT interval (p=0.024); (5) P axis (p=0.005); (6) QRS axis (p=0.012). The paediatric/young subgroup had significantly higher heart rate (p=0.041), while a greater proportion of adult patients suffers from interventricular delay (p=0.015).

### Spontaneous VT/VF predictors

Univariate Cox regression for postdiagnosis VT/VF-free survival demonstrated the following significant predictive variables (table 2): (1) family history of LQTS (HR=0.359, 95% CI (0.163 to 0.794), p=0.011); (2) initial QTc interval (HR=1.01, 95% CI (1.00 to 1.02), p=0.011); (3) positive treadmill test (HR=0.086, 95% CI (0.009 to 0.793), p=0.030); (4) occurrence of other arrhythmias (HR=4.50, 95% CI (2.32 to 8.74), p<0.0001); (5) Schwartz score (HR=1.49, 95% CI (1.08 to 2.05), p=0.015); (6) initially asymptomatic (HR 0.301, 95% CI (0.136 to 0.662), p=0.003); (7) initial presentation of VT/VF (HR=5.80, 95% CI (2.94 to 11.5), p<0.0001); (8) QRS interval (HR=1.03, 95% CI (1.01 to 1.04), p=0.001); (9) QTc interval (HR=1.02, 95% CI (1.01 to 1.02), p<0.001); (10) presence of interventricular delay (HR=3.11, 95% CI= (1.37 to 7.08), p=0.007). Similarly, the significant predictors from the adult subgroup include: (1) presence of other arrhythmias (HR=4.49, 95% CI (1.85 to 10.9), p=0.001); (2) Schwartz score (HR=3.76, 95% CI (1.70 to 8.34), p=0.001); (3) initially asymptomatic (HR=0.357, 95% CI (0.129 to 0.986), p=0.047); (4) initial presentation of VT/VF (HR=2.82, 95% CI (1.14 to 7.03), p=0.026); (5) QRS interval (HR=1.03, 95% CI (1.01 to 1.04), p=0.002); (6) the presence of interventricular conduction delay (HR=3.60, 95% CI (1.38 to 9.41), p=0.009).

**Table 2:** Univariate predictors of postdiagnosis VT/VF-free survival

Predictor	Overall			Adult		
	HR	95% CI	P value	HR	95% CI	P value
Female	1.13	(0.568 to 2.25)	0.730	1.37	(0.518 to 3.60)	0.529
Onset age	1.01	(0.997 to 1.02)	0.146	0.992	(0.963 to 1.02)	0.573
Family history of LQTS	0.359	(0.163 to 0.794)	**0.011**	0.427	(0.154 to 1.19)	0.103
Family history of SCD	0.148	(0.020 to 1.08)	*0.060*	0.162	(0.022 to 1.22)	*0.077*
Initial asymptomatic	0.301	(0.136 to 0.662)	**0.003**	0.357	(0.129 to 0.986)	**0.047**
Initial syncope	0.721	(0.345 to 1.50)	0.383	1.08	(0.411 to 2.83)	0.878
Initial VT/VF	5.80	(2.94 to 11.5)	**<0.0001**	2.82	(1.14 to 7.03)	**0.026**
Initial QTc interval	1.01	(1.00 to 1.02)	**0.007**	1.01	(0.997 to 1.01)	0.227
Positive treadmill test	0.086	(0.009 to 0.793)	**0.030**	/	/	/
Positive EPS	0.377	(0.039 to 3.63)	0.399	1.09	(0.089 to 13.3)	0.948
Arrhythmia in Holter Study	1.10	(0.330 to 3.67)	0.877	0.449	(0.075 to 2.69)	0.381
Other arrhythmias	4.50	(2.32 to 8.74)	**<0.0001**	4.49	(1.85 to 10.9)	**0.001**
Schwartz Score	1.49	(1.08 to 2.05)	**0.015**	3.76	(1.70 to 8.34)	**0.001**
Follow-up duration	0.999	(0.993 to 1.00)	0.634	0.999	(0.992 to 1.01)	0.787
Baseline ECG characteristics						
Heart rate	1.00	(0.987 to 1.02)	0.743	1.01	(0.983 to 1.03)	0.562
P-wave duration	0.988	(0.946 to 1.03)	0.571	0.998	(0.959 to 1.04)	0.932
PR interval	1.00	(0.985 to 1.02)	0.754	1.00	(0.981 to 1.03)	0.809
QRS interval	1.03	(1.01 to 1.04)	**0.001**	1.03	(1.01 to 1.04)	**0.002**
QT interval	1.00	(0.997 to 1.01)	0.359	1.00	(0.995 to 1.01)	0.500
QTc interval	1.02	(1.01 to 1.02)	**<0.001**	1.01	(1.00 to 1.02)	*0.062*
P axis	0.998	(0.988 to 1.01)	0.737	0.994	(0.982 to 1.01)	0.383
QRS axis	1.00	(0.995 to 1.01)	0.494	1.00	(0.994 to 1.01)	0.620
T axis	1.00	(0.994 to 1.01)	0.705	0.998	(0.990 to 1.01)	0.695
Lead V5 R wave amplitude	1.15	(0.587 to 2.26)	0.680	0.761	(0.371 to 1.56)	0.456
Lead V1 S wave amplitude	2.09	(0.699 to 6.26)	0.187	1.73	(0.556 to 5.37)	0.344
First degree AV block	1.92	(0.440 to 8.39)	0.386	3.91	(0.770 to 19.9)	0.100
Interventricular delay	3.11	(1.37 to 7.08)	**0.007**	3.60	(1.38 to 9.41)	**0.009**

EPS, electrophysiological study; LQTS, long QT syndrome; LQTS, long QT syndrome; SCD, sudden cardiac death; VT/VF, ventricular tachycardia/ventricular fibrillation.

**Figure 1:**
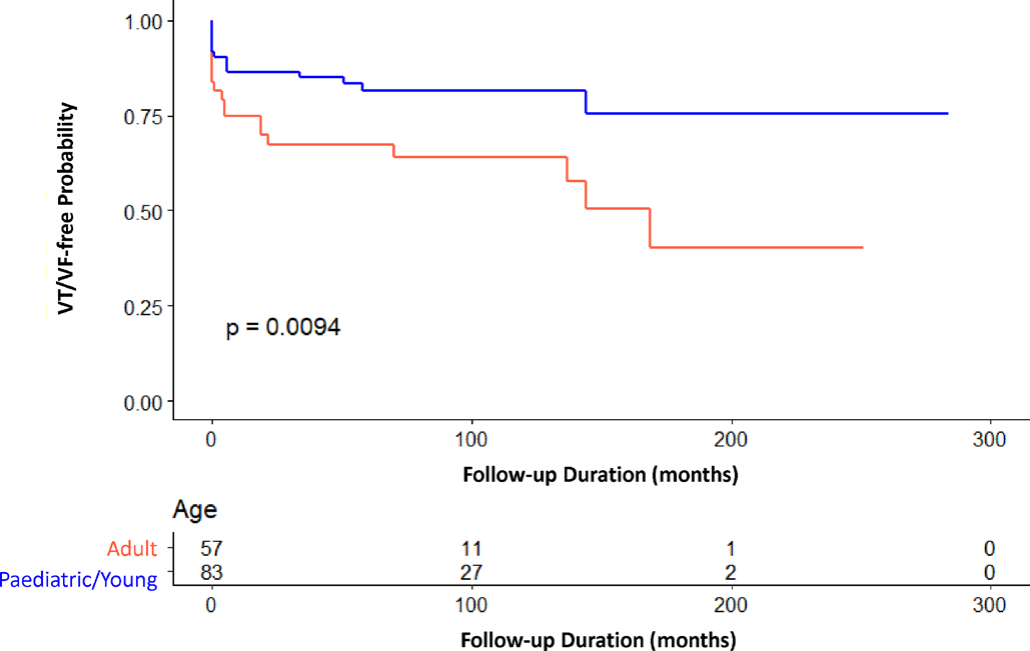
Kaplan-Meier survival curve for paediatric/young and adult long QT syndrome patients. VT/VF, ventricular tachycardia/ventricular fibrillation.

The detailed findings from the multivariate Cox regression analysis are shown in tables 3 and 4. Additionally, paediatrics patients were found to have significantly longer VT/VF-free postdiagnosis (HR=0.425, 95% CI= (0.217 to 0.832), p=0.013). Figure 1 displays the significant intergroup difference in the Kaplan-Meier survival curve (p=0.009).

**Table 3:** Multivariate predictors of postdiagnosis VT/VF-free survival baseline ECG parameters

Overall (n=136)				Adult (n=58)			
Parameter	HR	95% CI	P value	Parameter	HR	95% CI	P value
Family history of LQTS	0.750	(0.330 to 1.71)	0.492	Other arrhythmia	3.08	(1.27 to 7.49)	**0.013**
Other arrhythmia	3.09	(1.60 to 6.00)	**<0.0001**	Initial VT/VF	2.58	(1.05 to 6.35)	**0.039**
Initial VT/VF	4.20	(2.03 to 8.67)	**<0.001**	Schwartz score	3.73	(1.63 to 8.52)	**0.002**

LQTS, long QT syndrome; VT/VF, ventricular tachycardia/ventricular fibrillation.

**Table 4:** Multivariate predictors of postdiagnosis VT/VF-free survival including baseline ECG parameters

Overall (n=88)				Adult (n=44)			
Parameter	HR	95% CI	P value	Parameter	HR	95% CI	P value
Family history of LQTS	0.843	(0.315 to 2.25)	0.734	Other arrhythmia	2.64	(1.02 to 6.83)	**0.046**
Other arrhythmia	3.06	(1.39 to 6.75)	**0.006**	Initial VT/VF	2.48	(0.945 to 6.48)	0.065
Initial VT/VF	2.86	(1.31 to 6.26)	**0.009**	Schwartz score	3.68	(1.33 to 10.2)	**0.012**
Initial QTc interval	1.01	(0.996 to 1.02)	0.255	QRS Interval	1.02	(1.01 to 1.04)	**0.005**
QRS interval	1.02	(1.00 to 1.04)	**0.019**				

LQTS, long QT syndrome; VT/VF, ventricular tachycardia/ventricular fibrillation.

### Survival analysis using machine learning

**Figure 2:**
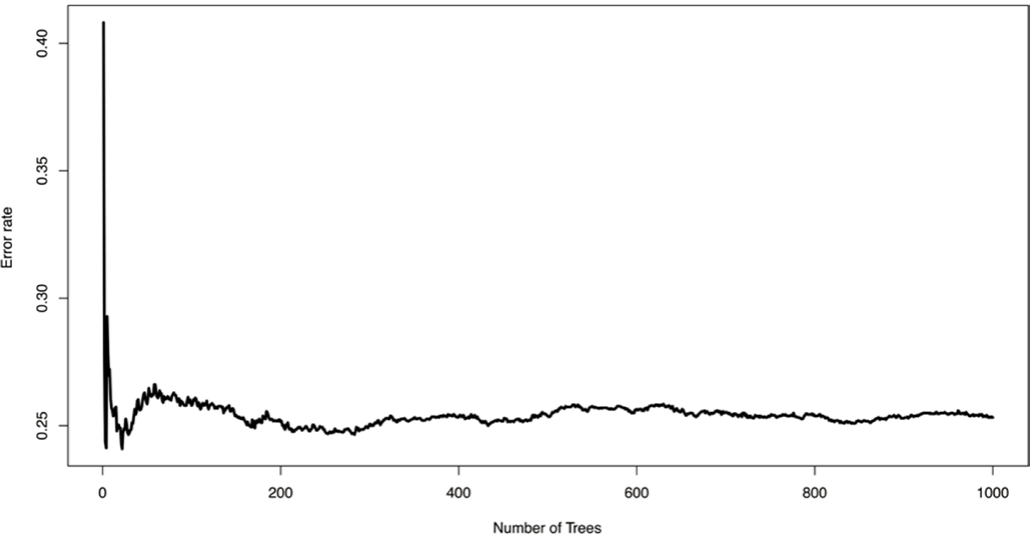
Optimal tree number selection in the random survival forest model for predicting ventricular tachycardia/ventricular fibrillation.

**Figure 3:**
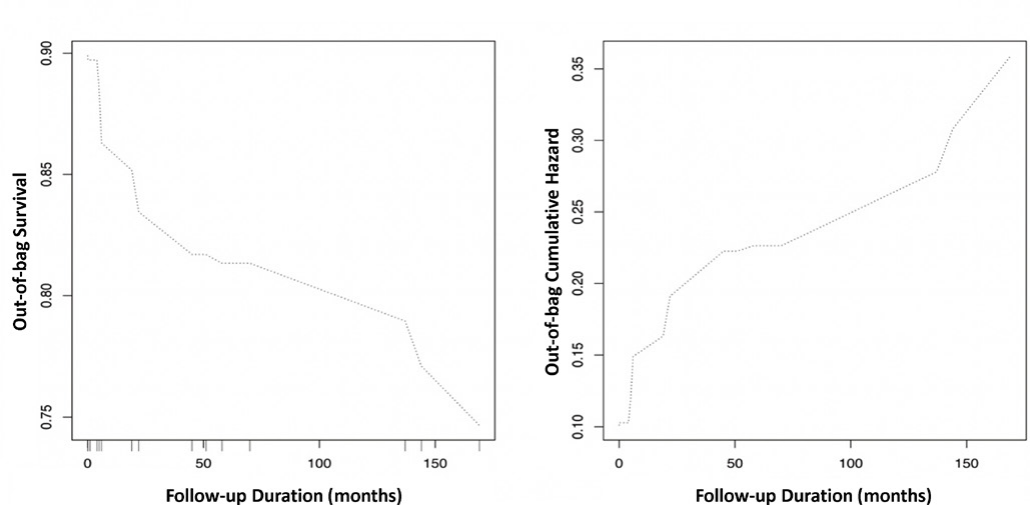
Predicted out-of-bag survivals and cumulative hazards generated by the random survival forest model for predicting ventricular tachycardia/ventricular fibrillation.

The application of RSF to the cohort data yielded the importance ranking of significant risk predictors (table 5). Initial VT/VF was identified as the most important variable to predict VT/VF outcome, followed by Schwartz score, initial QTc interval, family history of LQTS, initially asymptomatic status and the presence of arrhythmias other than VT/VF. By contrast, the family history of SCD provided a limited predictive strength. The optimal tree number of RSF to predict VT/VF was set to 200 (figure 2). The predicted out-of-bag survivals and cumulative hazards generated by the RSF model are shown in figure 3.

**Table 5:** Importance ranking of significant univariate predictors of incident VT/VF generated by the RSF model

Predictor	Variable importance	Rank
Initial VT/VF	0.1006	1
Schwartz Score	0.0370	2
Initial QTc Interval	0.0303	3
Family history of LQTS	0.0054	4
Initially asymptomatic	0.0028	5
Other arrhythmias	0.0002	6
Family History of SCD	−0.0010	–

LQTS, long QT syndrome; RSF, random survival forest; SCD, sudden cardiac death; VT/VF, ventricular tachycardia/ventricular fibrillation.

**Figure 4:**
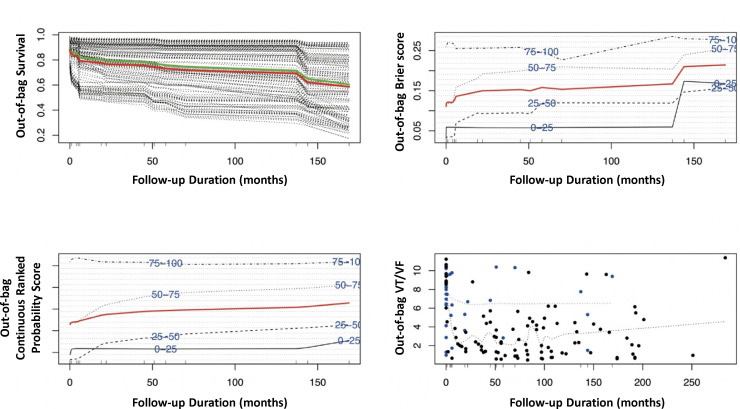
Survival estimates generated from the random survival forest model. The overall ensemble survival is indicated by the red line; the Nelson-Aalen estimator is given by the green line (top left panel). Brier score (0=perfect, 1=poor and 0.25=guessing) stratified by ensemble mortality based on the inverse probability of censoring weight method (top right panel). The cohort was stratified into four groups of 0–25, 25–50, 50–75 and 75–100 percentile mortality (the overall, non-stratified, Brier score is shown by the red line). Continuous Rank Probability Score given by the integrated Brier score divided by time (bottom left panel). Plot of incident VT/VF of each LQTS patient versus observed time (bottom right panel). Events are shown as blue points, whereas censored observations are shown as red points. LQTS, long QT syndrome; VT/VF, ventricular tachycardia/ventricular fibrillation.

**Figure 5:**
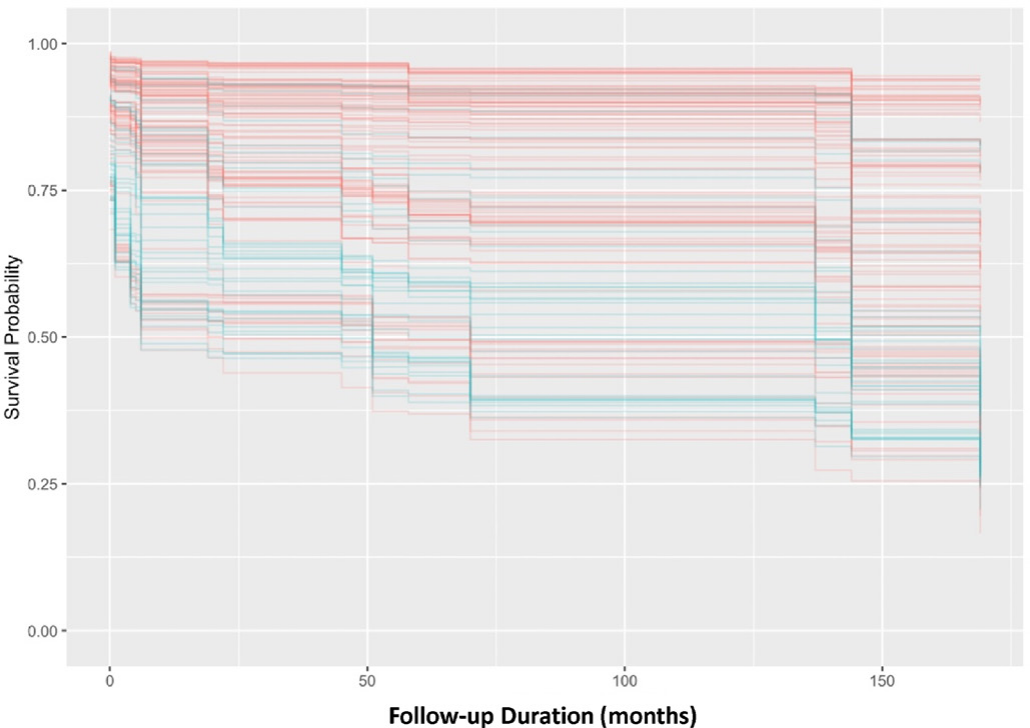
Predicted ventricular tachycardia/ventricular fibrillation survival generated by the random survival forest model.

The survival functions estimated for each patient with the RSF model to predict VT/VF are shown in figure 4. The overall ensemble survival is indicated by the red line, whereas the Nelson-Aalen estimator is given by the green line figure 4 (top left panel). Brier score (0=perfect, 1=poor, and 0.25=guessing) stratified by ensemble VT/VF based on the IPCW method is shown in the figure 4 (top right panel). The cohort was stratified into four groups of 0–25, 25–50, 50–75 and 75–100 percentile VT/VF (the overall, non-stratified, Brier score is shown by the red line). A Continuous Rank Probability Score given by the integrated Brier score divided by time is shown in the bottom left panel, whereas a plot of VT/VF of each individual versus observed time is shown in the bottom right panel. Events are shown as blue points, whereas censored observations are shown as red points. The estimates of survival probability generated by the RSF model are provided in figure 5.

Finally, the predicted VT/VF survival using the RSF model are shown in figure 5, respectively. The blue curves correspond to censored observations while the red curves correspond to observations experiencing VT/VF events. The survival analysis performance of the RSF model was compared with that of the multivariate Cox model to predict the next VT/VF outcome of patients in a five-fold cross-validation approach (table 6). The RSF model significantly outperformed the multivariate Cox model (precision: 0.95, recall: 0.93, Brier score: 0;.09, Harrell’s C-index: 0.91) based on the same inputs of significant univariate predictors.

**Table 6:** Performance comparisons of RSF and multivariate COX models to predict VT/VF (both with fivefold cross-validation approach and significant univariate predictors as model input)

Model	Precision	Recall	Brier score	Harrell’s C index
RSF	0.95	0.93	0.09	0.91
Multivariate cox	0.86	0.84	0.13	0.82

RSF, random survival forest; VT/VF, ventricular tachycardia/ventricular fibrillation.

## Discussion

This is the first population-based cohort study from Hong Kong comparing paediatric/young and adult patients suffering from LQTS. There are several major findings for the present study: (1) there are significant differences in clinical and ECG presentation among adult and paediatric/young LQTS patients; (2) adult LQTS patients have a higher risk for spontaneous VT/VF; (3) similar predictors were found in the overall LQTS cohort and adult subgroup; (4) a non-parametric machine learning survival analysis can achieve much higher accuracy to predict the incident VT/VF probabilities of LQTS patients through accounting for the interactions between predictors.

In terms of disease manifestation, an initial presentation with syncope was more common in the paediatric/young compared with the adult patients (39% vs 22%), whereas initial presentation of VT/VF was more common in adults than paediatric/young patients (36% vs 14%). Overall, the adult subgroup carried a greater risk for VT/VF occurrence and all-cause mortality. This can be explained by early beta-blocker use among paediatrics patients, and the difference in age of peak VT/VF risk between different subtypes of LQTS. For example, while patients are most susceptible to spontaneous VT/VF throughout childhood for type 1 LQTS, postpartum females are most vulnerable in type 2 LQTS, and mortality risk is elevated from ages 10 to 59 years in syndromes with SCN5A mutation.^6 26^ The effectiveness of beta-blocker in SCD risk reduction among LQTS patients, particularly the paediatric population, is well demonstrated.^27^ Due to the difficulty in VT/VF risk prediction among young LQTS patients, prophylactic beta-blockers are prescribed in the absence of contraindications.^28^ Given that non-compliance is a major cause for treatment failure in beta-blocker use, it is speculated that the paediatric/young subgroup may have better compliance with the involvement of parental efforts to reinforce the drug compliance.^28 29^ Surprisingly, a family history of LQTS was found to be protective against VT/VF occurrence during follow-up under univariate analysis, which may be attributed to earlier disease diagnosis and treatment intervention. Additionally, unlike most parts of the world, the lack of routine family screening programmes in Hong Kong prevents the diagnosis of asymptomatic children. The underestimation of asymptomatic cases results in a staggeringly high proportion of patients with VT/VF during follow-up.

Although baseline QTc interval was only predictive of spontaneous VT/VF under univariate analysis, Schwartz score, a diagnostic score of LQTS that accounts for QTc interval and other clinical parameters remained predictive in multivariate analysis. The predictiveness of the Schwartz score suggests that a combination of clinical and ECG indices should be considered holistically in the risk stratification of LQTS. Furthermore, while the value of diagnostic criteria in hereditary LQTS is questioned for its low sensitivity in the era of molecular testing, the present finding demonstrates the potential application value of diagnostic criteria in risk stratification.^30^ Additionally, the protective value of positive treadmill results can be explained by that the treadmill stress test is only used in diagnosis for borderline patients, which are inherent of lower risk. Patients of severe phenotype would not have undergone the treadmill stress test, therefore, resulted in the falsely protective nature of positive treadmill test results.^31^ Interestingly, 24-hour Holter monitoring and treadmill tests were more frequently performed in the paediatric/young subgroup compared with the adult subgroup in LQTS. We speculate that this may reflect differences in clinical practice for paediatric in comparison to adult cardiologists.

The significant intergroup differences in ECG indices can be attributed to both the inherent cardiac electrophysiological differences between adult and paediatric/young patients and LQTS-specific age-dependent differences. Increased heart rate, right-sided QRS axis deviation and shortened PR interval among paediatric patients, in comparison to adults, is normal.^32^ Old age is associated with longer PWD with an increased likelihood of abnormal P-wave axis due to regional electroanatomical changes in the atrium.^33–35^ Furthermore, QRS duration lengthens over increased age with an elevated risk for intraventricular conduction delays, likely due to the age-dependent risk of ischaemic heart disease and metabolic diseases, which can affect the His-Purkinje system.^36 37^ The association between T-wave axis deviation, hypertension and diabetes mellitus supports this hypothesis.^38^

There are few studies on ECG changes aside from QTc interval and T-wave abnormalities in LQTS and the potential age-dependent variations in patients’ ECG profile have yet to be explored. Atrial enlargement was found to be significantly more common among older patients.^39^ It has been reported that the use of vectorcardiogram improves the accuracy of QT interval determination in children, which may be explained by the greater ability of vectorcardiogram to capture the cardiac axis that possibly deviates from the norm.^40^ Further research is needed to explore the age-dependent difference in ECG profiles of LQTS patients and its potential application in risk stratification. The occurrences of atrial arrhythmias and spontaneous VT/VF are closely linked. Altered atrial electrophysiology is found in LQTS, with a similar arrhythmic substrate of delayed repolarisation occurring in both the atria and the ventricles.^41 42^ The shortening of the diastolic interval during atrial tachyarrhythmia can occasionally lead to greater spatial dispersion of action potential duration, which can produce the ventricular substrate that promotes reentry.^43–45^

### Strengths and limitations

The major strengths of the present study include (1) predictors of postdiagnosis VT/VF-free survival were derived for adult and paediatric/young patients; (2) holistic differences in clinical and ECG aspects of adult and paediatric/young patients were evaluated; (3) the study cohort was followed-up for a substantial length of time and (4) the use of machine learning algorithms, which was previously used by our team,^46^ to improve risk prediction.

Several limitations should be noted for the present study. First, the retrospective nature of the study is inherently subjected to selection and information bias. However, consultations were performed at least annually for most patients, hence the patients were closely followed up. Also, it should be noted that the documented syncope may not be of cardiogenic origin, hence it may be unrelated to the ion channelopathy. Data on the type of ICDs implanted were not available for all patients and thus it was not possible to summarise them for the cohort. Furthermore, changes in guidelines for investigations and diagnostic tests throughout follow-up introduced inevitable inconsistency in indications for different tests. Additionally, although this is a territory-wide registry, the strikingly low prevalence of LQTS in Hong Kong is due to the combination of undercoding and a lack of an effective family screening programme. As a result, the number of asymptomatic LQTS patients is grossly underestimated.

Due to the limited availability of public genetic services, not all patients with ion channelopathies have undergone genetic screening, and hence genotype–phenotype correlations could not be established with greater degrees of certainty. Genetic testing has evolved over the past years. Before 2014, testing was limited to a panel of six genes for LQTS (KCNQ1, KCNH2, SCN5A, KCNE1, KCNE2, KCNJ2), and sequencing of exon hotspots for CPVT (exons 1, 8, 14, 15, 44, 46, 47, 49, 88, 93, 95, 97, 101, 102, 103, 104, 105). After 2014, next-generation sequencing (NGS) was offered. We recognise the differences in methodology make the interpretation difficult, and we cannot exclude the possibility of false-negative if NGS was applied to all cases. However, the retrospective nature review of case notes means that we are not able to elucidate this further in our study. Future studies should apply NGS to all patients to identify additional gene mutations responsible for LQTS.

## Conclusion

Clinical and ECG presentation of LQTS vary between the paediatric/young and adult LQTS population. Risk stratification and management strategies against young patients should take into consideration the difference between paediatric and adult patients and adopt an individualised approach. Machine learning models achieved more accurate VT/VF prediction.

## Data Availability

Data are available in a public, open access repository. Data are available on reasonable request.
